# Comment on Küppers et al. Percutaneous Anorectoplasty (PARP)—An Adaptable, Minimal-Invasive Technique for Anorectal Malformation Repair. *Children* 2022, *9*, 587

**DOI:** 10.3390/children9091381

**Published:** 2022-09-14

**Authors:** Alberto Peña

**Affiliations:** International Center for Colorectal and Urogenital Care, Division of Pediatric Surgery Children’s Hospital, Department of Surgery, Division of Pediatric Surgery, School of Medicine, University of Colorado, 13213 E 16th Ave, Box 323, Anschutz Medical Campus, Aurora, CO 80045, USA; penabischoffcolped@gmail.com

I read the above-mentioned article with a great deal of interest.

The authors operated on ten patients—five of them with a perineal fistula and five without evidence of a fistula—using the technique that they called ‘PARP’ [1].

Attempts to innovate surgical techniques to improve results in the management of anorectal malformations are always welcomed. The authors demonstrated a great deal of creativity and imagination and were able to take advantage of sophisticated technological advances to pursue their goals.

I believe it is worthwhile to make a few key observations:

The surgical technique used by us to repair perineal fistulae is known as “mini-PSARP”. It is, by definition, a minimally invasive operation, it takes about 90 min to complete, the patients experience no postoperative pain, and one hundred percent of them achieve bowel control by the age of 3 years (except when there is an abnormal sacrum, a hemi sacrum, or a sacral ratio of less than 0.4). Most of the patients will suffer from constipation, which is medically manageable.

In patients suffering from anorectal malformations without a fistula, we perform a Posterior Sagittal Anorectoplasty (PSARP). Ninety percent of patients without Down syndrome have bowel control by the age of 3 years. Eighty percent of those with Down syndrome achieve bowel control by the age of 3 years, provided that they have a normal sacrum. In Down syndrome cases, it is important to remember that patients suffer from different degrees of developmental delay, which influences their ability to achieve bowel control.

I was surprised by the claim made by the authors of this paper that all of their patients were fecally continent, including one with Currarino syndrome. I was further perplexed to learn that the evaluation of bowel control was conducted when the patients were 2, 6, 8, 9, 15, 16, 20, 23, and 27 months old. Only one patient was older than three years old. This raises a very serious question about the evaluations of the patients: Are the authors claiming that the babies ranging in age from 2 to 16 months had bowel control? If true, then this would be more advanced than normal children. How do the authors define ‘bowel control’? We define bowel control as the “capacity of the patients to verbalize the need to evacuate their bowel and their ability to maintain their underwear completely clean 24 h per day”. We evaluate bowel control in our patients when they reach the age of three, specifically because this is the age at which the great majority of normal children gain bowel control.

Another apparent omission is the fact that the authors did not mention the characteristics of the sacrum in their patients, which is the most important prognostic factor in anorectal malformations. Perineal fistulae, vestibular fistulae, recto-urethral bulbar fistulae, and malformations without a fistula, with a normal sacrum, are the cases that benefit the most from a technically correct PSARP and they represent the most favorable end of the spectrum.

The pediatric surgical community, the unfortunate patients suffering from anorectal malformations, and their families are all eagerly awaiting advancements in technology and treatments that will improve bowel control for the children suffering from the most challenging forms of these malformations. At the present time, the real challenges that we are facing with children (with normal sacrums) who are treated with PSARP are as follows.

My suggestion to the new generation of pediatric surgeons who are eager to make a significant contribution to the management of anorectal malformations is to focus on what, in my experience, have proven to be the most challenging defects, including:

− Recto-urethral prostatic fistula: a 60% chance of bowel control by the age of three years.
− Recto-bladder-neck fistula: a 20% chance of bowel control by the age of three.
− Cloacas with a common channel longer than 3 cm: a 70% chance of bowel control.

We are all cheering for the new generation of pediatric surgeons to tackle these challenges in order to elevate the overall prognosis for children born with these malformations.

Thank you for your time and attention.

## References

[1] Küppers J., van Eckert V., Muensterer N.R., Holler A.-S., Rohleder S., Kawano T., Gödeke J., Muensterer O.J. (2022). Percutaneous Anorectoplasty (PARP)—An Adaptable, Minimal-Invasive Technique for Anorectal Malformation Repair. Children.

